# Epidemiological Study of *Mycobacterium bovis* Infection in Buffalo and Cattle in Amazonas, Brazil

**DOI:** 10.3389/fvets.2019.00434

**Published:** 2019-12-10

**Authors:** Paulo A. M. Carneiro, Haruo Takatani, Taynara N. Pasquatti, Christian B. D. G. Silva, Bo Norby, Melinda J. Wilkins, Martín José Zumárraga, Flabio R. Araujo, John B. Kaneene

**Affiliations:** ^1^Center for Comparative Epidemiology, College of Veterinary Medicine, Michigan State University, East Lansing, MI, United States; ^2^Amazonas State Federal Institute, Manaus, Brazil; ^3^Agência de Defesa Agropecuaria do Amazonas, Manaus, Brazil; ^4^Dom Bosco Catholic University, Campo Grande, Brazil; ^5^Department of Large Animal Clinical Sciences, College of Veterinary Medicine, Michigan State University, East Lansing, MI, United States; ^6^Institute of Biotechnology, CICV/INTA, Buenos Aires, Argentina; ^7^Centro Nacional de Pesquisa de Gado de Corte, Campo Grande, Brazil

**Keywords:** bovine tuberculosis, *Mycobacterium bovis*, zoonosis, cattle, buffalo, epidemiology

## Abstract

Bovine Tuberculosis (BTB) is an endemic disease in about one hundred countries, affecting the economy causing a decrease in productivity, condemnation of meat, and damaging the credibility on international trade. Additionally, *Mycobacterium bovis* the major causative agent for BTB can also infect humans causing a variety of clinical presentations. The aim of this study was to determine BTB prevalence and the main risk factors for the *Mycobacterium bovis* prevalence in cattle and buffalos in Amazonas State, Brazil. Tissue samples from 151 animals (45 buffalo and 106 cattle from five herds with buffalo only, 22 herds with cattle only, and 12 herds with buffalo and cattle) were obtained from slaughterhouses under State Veterinary Inspection. *M. bovis* were isolated on Stonebrink medium. The positive cultures were confirmed by polymerase chain reaction (PCR) testing. The apparent herd and animal prevalence rates were 56.4 and 5.40%, respectively. Regarding animal species, the apparent prevalence rates were 3% in cattle and 11.8% in buffalo. Generalized Linear Mixed Models (GLMM) with random effect were used to assess the association with risk factors on the prevalence. Species (buffalo), herds size (>100 animals) and the presence of both species (buffalo and cattle) in the herd were the major risk factors for the infection by *Mycobacterium bovis* in the region. The findings reveal an urgent need for evidence-based effective intervention to reduce BTB prevalence in cattle and buffalo and prevent its spread to the human population. Studies are needed to understand why buffalo are more likely to be infected by *M. bovis* than cattle in Amazon. Recommendations for zoning, use of data from the inspection services to generate information regarding BTB focus, adoption of epidemiological tools, and discouragement of practices that promote the mixing of cattle and buffalo, were made.

## Introduction

Bovine tuberculosis (BTB) remains one of the world's major health problems in livestock. BTB affects the national economy of countries where disease is endemic by causing a decrease in productivity, condemnation of meat in slaughterhouses, and decreasing the ability for international trade (1). During 2015 to 2016, 179 countries reported the presence of the disease in livestock and/or wildlife, demonstrating its wide geographical distribution (2).

*Mycobacterium bovis (M. bovis)* is the causative agent of BTB and is also responsible for the zoonotic tuberculosis (TB) which is a major impediment for the success of the global efforts to end TB by the year 2030 (3). Although estimates of the global burden of zoonotic TB are imprecise, in 2016 WHO estimated that there were 147,000 new cases of zoonotic TB in humans and 12,500 deaths due to the disease (4). The human burden of disease cannot be reduced without controlling BTB in the animal reservoirs (4).

In many industrialized countries, the implementation of national BTB programs, based on regular tuberculin testing and removal of infected animals, had led to the successful eradication or a major reduction in the incidence of BTB in cattle herds (5). However, these control measures have been only partially effective in countries or regions with a wildlife reservoir of infected animals, such as the United Kingdom (UK), New Zealand and the United States of America (USA) (6–8). Furthermore, these measures are not affordable in most countries of the world, particularly in countries which have a high prevalence of BTB in their domesticated livestock population (9).

In Brazil, the National Program for the Control and Eradication of Brucellosis and Bovine Tuberculosis (PNCEBT) was establish in 2004 and is based on the sacrificing of all animals displaying positive reaction to tuberculosis tests (10). In recent years epidemiological studies were conducted to determine the BTB status in several Brazilian states (11–24), however no studies were conducted in Amazonas State. Moreover, a detailed understanding of the risk factors involved in the *M. bovis* transmission is an identified gap in BTB studies. Understanding the epidemiology of the disease is fundamental for the development of efficient disease control strategies (9, 25).

Statistical modeling studies are important to elucidate the transmission dynamics of BTB within and between herds (26–29). Additionally, mathematical modeling studies have been carried out to analyze disease transmission and provide insight into useful control measures (30–33).

This study aims to ascertain the prevalence of BTB and, through statistical modeling, unveil the main risk factors of the disease in cattle and water buffalos in Amazonas State, Brazil. Ultimately our goal is to propose evidence-based measures to improve the regional programs for the eradication of TB caused by *M. bovis* in livestock and humans.

## Materials and Methods

### Study Population

In Amazonas State, cattle and buffalo, are predominantly managed in extensive and semi-confined systems, there are no herds raised in a total confined system. In an extensive system, animals remain in the pasture most of the time and the feeding system is based strictly on grazing with mineral salt being offered in feeders on the pasture. Herd health is based on palliative care of animals that present wounds or signs of illness, and the preventive care is restricted to semi-annual vaccination of Foot and Mouth Disease. Cattle are predominantly mixed *Bos indicus* or mixed *Bos taurus indicus*; buffalo are predominantly mixed breeds Murrah, Carabao, and Mediterranean. In semi-confined models, animals are gathered daily in pens where food supplementation and mineral salt are provided in separate feeders. Within the Semi-confined systems, herd health is more appropriate, animals are observed daily for injuries or signs of illness, the preventive care usually is composed of control of parasites, vaccination against Foot and Mouth Disease and Brucellosis. Cattle are predominantly of the Nelore and Girolando breeds, for beef and dairy, respectively. Buffalo are predominantly Murrah (dairy and beef) and Mediterranean (dairy).

In common, the husbandry systems of the two species are influenced by flooding during the raining season. During the rainy season (November to June) herds remain at the mainland areas. During the dry season (July to mid-November) weaned calves, steers, heifers, and dry cows are transported to shared floodplain grassland for beef or recovery purpose. Apui is the only municipality in this study not influenced by flooding.

Buffalo and cattle are raised adopting the same management farming system, but due to having more resistance to flooding in regard hoof problems, buffalos are moved from the mainland to the floodplains earlier, and moved back later, than cattle. On average, buffalos spend an additional 3 months in floodplains compared to cattle.

Herds (*n* = 39) from three intermediary regions and 13 municipalities were included on this study. Twenty-two herds (56.4%) were composed only by cattle, 12 herds were composed of buffalo and cattle (30.8%), and five (12.8%) herds were composed only by buffalo. The total number of animals inspected during the sampling were 832 (229 buffalo and 603 cattle), and from those 151 samples tissues (45 buffalo and 106 cattle) were obtained (Table 1). The median age group of inspected animals in both species were from 25 to 36 months old, and the mean herd size was 142 for cattle and 84 for buffalos.

**Table 1:** Distribution of the sampling by origin, number of animals inspected, sample by species, and percent of the sampling, Amazonas State, Brazil.

**Region**	**Municipality**	**Animals inspected**	**Buffalo**	**Cattle**	**%**
Labrea	Apui	122	0	26	17.22
	Manicore	19	0	1	0.66
	Novo Aripuana	83	0	14	9.27
Manaus	Autazes	108	24	2	17.22
	Careiro	24	1	1	1.32
	Careiro da Varzea	121	0	14	9.27
	Iranduba	7	0	4	2.65
	Manacapuru	98	0	4	2.65
	Manaquiri	6	0	6	3.97
	Pres. Figueiredo	80	0	26	17.22
Parintins	Itacoatiara	56	2	8	6.62
	Parintins	50	9	0	5.96
	Urucara	58	9	0	5.96
TOTAL		832	45	106	100.00

Of all the animals in the study, 48.3% were from small size herds, 28.4% from medium herds, and 23.1% from large herds. Additionally, 82.7% of the animals were from farms with herds of only one specie (cattle or buffalo). With regard to the purpose, 49.6% of the animals were from herds with mixed purpose, beef and dairy animals represented 31.1 and 19.2% of the sampling, respectively (Table 2).

**Table 2:** Description and descriptive statistics for animal-level risk factors evaluated for 151 animals (106 cattle and 45 buffalo) in 39 herds in Amazonas State.

**Risk factor^**a**^**	**Description**	***N***	**%**
Specie	Cattle	106	70.20
	Buffalo	45	29.8
Herd size	Small	73	48.34
	Medium	43	28.48
	Large	35	23.18
Herd age	≤12 months	0	0
	13–24 months age	42	27.81
	24–36 months age	54	35.76
	≥36 months	55	36.42
Cattle and buffalo	No	125	82.78
	Yes	26	17.22
Farming system	Confined	0	0
	Semi-confined	79	52.32
	Extensive	72	47.68
Purpose	Beef	47	31.13
	Dairy	29	19.21
	Mix	75	49.67
Habitat	Floodplains	71	47.02
	Mainland	80	52.98
History	No	129	85.43
	Yes	22	14.57
Herd health	No	46	30.46
	Yes	105	69.54

a *Admitted to the starting multivariable model because it passed screening (p < 0.50)*.

### Criteria for Inclusion

The study was based on a convenience sampling of adult animals sent for commercial slaughter at three major slaughterhouses in Amazonas State.

From herds with a report of the official tuberculin skin test (TST) performed and reactive buffalo or cattle, samples of all animals sent to the slaughterhouses, with or without Lesion Suggestive of Tuberculosis (LST), were collected. The Caudal Fold Test (CFT), the Simple Cervical Test (SCT), and the Comparative Cervical Test (CCT) are the official tests of detection. The CFT and SCT were adopted as screening tests for beef and dairy cattle, respectively, while the CCT was adopted as a confirmatory test for animals positive at the screening test (5). From herds with unknown TST status, samples were collected from all animals with visible tubercles and from animals with suspicious granulomatous lesions.

The inspection of the animals was performed by State Veterinary Inspection Service (SIE) trained officials, LST were defined as granulomas a mass or nodule of chronically inflamed tissue, yellow or tan, and either caseous, caseo-calcareous or calcified. The same criteria for detection of lesions were used for cattle and buffalo.

### Study Design

This study is a cross-sectional study performed from July of 2016 to February of 2018. Two samples per animal were collected, one from the suspicious tissue and other from the respiratory system lymph nodes found with increase of volume or LST or from the medial retro-pharyngeal lymph nodes in case of no alterations found in lymph nodes. The option for the medial retro pharyngeal lymph nodes is based on our experience in Michigan (34). The unit of analysis was the animal. The individual animal was considered BTB positive if the culture growing was confirmed by the polymerase chain reaction (PCR) as *M. bovis*, in either tissue samples. For the herd-level analysis, the herd was considered infected when it presented at least one animal confirmed positive by the PCR analysis. The animals were slaughtered for commercial purposes, there were no animals sacrificed due to this study.

### Preparation and Culture of Samples

Lesions from suspected animals (10–25 mg) were processed and inoculated in duplicate into Stonebrink medium (35). The Stonebrink medium has the same composition as Löwenstein–Jensen, except that glycerol is replaced by 0.5% sodium pyruvate, further incubated at 37°C and evaluated weekly for 90 days to verify bacterial growth. One medium per sample were used and the colonies with characteristics suggestive of *M. bovis* were submitted fur DNA extraction.

### DNA Extraction

The bacterial colonies were washed with 500 μL of Tris-EDTA (TE) buffer in micro-tubes and inactivated in a dry bath for 1 h at 87°C, with subsequent centrifugation at 14,000 rpm for 2 min. The pellet that formed was discarded and the supernatant containing the mycobacterial DNA was transferred to new micro-tubes and stored at −20°C for subsequent analysis.

### Microorganism Identification by PCR

The mycobacterial DNA samples were submitted to standard PCR according to Sales et al. (36), using primers Mb.400.F (5′AACGCGACGACCTCATATTC3′) and Mb.400.R (5′AAGGCGAACAGATTCAGCAT3′), which amplify a 400 base pair (bp) DNA fragment flanking the region of differentiation 4 (RD4), specific to *M. bovis* (37). The PCR products were stained with Gel Red and submitted to 1% agarose gel electrophoresis in 1X TAE buffer and visualized in a PhotoDocumentor under ultraviolet light.

### Sample Size

The sample size should be determined based on expected prevalence in samples from slaughterhouses, however, we are unable to find a previous study with this sample source in the region. In Amazonas state, only one study about prevalence of BTB in buffalos (*Bubalus bubalis*) was found, based on comparative cervical test (CCT) showing a prevalence of 20.4% (38). Recent studies about BTB prevalence in the region, also based on CCT, showed results ranging from 0.12 (cattle) to 7.2% (buffalos) in Rondonia and Para State, respectively (24, 39). Thus, as this study is based on a convenience sampling of cattle and buffalo, an expected prevalence of 10% was used for sample size calculations. With a test sensitivity of 97%, Type I error of 0.5%, and power of 80%, the minimum sample size needed was 139 animals.

### Risk Factors

The risk analysis was based on data obtained directly from the Animal Transportation Guide (GTA) and secondary data provided by the Amazonas State Animal Health Agency (ADAF). From each carcass sampled, epidemiological information, such as: origin, specie (cattle or buffalo), herd size, herd age, presence in the farm of both species, farming system, purpose, habitat, herd history of TB, and presence or absence of regular herd health practices, was collected.

Origin was defined by the municipality described on the GTA mandatory for the movement of animals from the farm to slaughterhouses. The species involved were cattle and water buffalo (*Bubalus bubalis*), the last raised in the region as livestock for the same purposes as cattle. Herd size was divided into three categories: (1) Small, herds ≤ 99 animals, (2) Medium, herds from 100 to 199 animals, and (3) Large herds with more than 200 animals. The same criteria were used for cattle and buffalo. Herd age was divided in 4 categories: (1) Animals ≤ than 12 months, (2) Animals, 13–24 months age, (3) Animals, 24–36 months age, and (4) Animals older than 36 months. The median age rank of the herd was used for the analysis. The study looked at the species composition of the herd, classifying if the herd is composed only of cattle, only of buffalo or a mix of cattle and buffalo.

Farming systems were divided in three categories: (1) Extensive, characterized by farms with mixed breed herds, low technological level and productivity, (2) Semi-confinement, characterized by farms with a predominant breed, adequate technological level and productivity, and (3) Confinement, characterized by farms with well-defined breeds, specialized for beef or dairy, excellent technical level and productivity.

The purpose of the farm was classified as adopted by ADAF as, Dairy—farms with the main activity to produce milk; Beef—farms of beef cattle; and Mix—farms without mainly objective defined, either can be dedicated to beef, in full or partial cycle (breading, rearing, and fattening) and to produce milk. In mixed farms, beef and dairy animals share environments and facilities.

Regarding the habitat, farms were classified according to the grazing area of the animal. In the Amazon region, herds can be moved between two ecosystems according the river flooding: The floodplains areas flooded during a 6-month period characterized by natural pastures of high nutritional value and the mainland areas not under influence of the rivers and characterized by artificial pasture planted after the removal of the native vegetation. Cattle and buffalo herds were classified according to the exposure to Floodplain grazing.

Based on secondary data from ADAF, animals were classified according to the historic presence or absence of BTB in their herds of origin. As the Brucellosis State Program requires vaccination of heifers which can only be done under veterinary supervision, herds with a register of vaccination were classified as having regular veterinary assistance, otherwise they were classified as not having regular herd health.

### Statistical Analysis of Expected Data

The prevalence was calculated by counting the data (animals *M. bovis* positive) per the reference population during the period of the outcome, according to method described by Dohoo et al. (40).

Given the nature of the outcome and number of risk factors, a multi-variable logistic regression model and a Generalized Linear Mixed Model (GLMM) with random effect was used to assess the influence of the risk factors on the prevalence, using 95% confidence intervals (*P* ≤ 0.05).

A summary of statistics was computed for each of the risk factors of interest (SAS^®^ 9.4, SAS Institute Inc., Cary, NC, USA). Univariable logistic regression for distinguishable data was conducted for each of the risk factors to assess their degree of association with the outcome variable (41).

The risk of *M. bovis* infection was evaluated using logistic regression for distinguishable data. The dependent variable (*M. bovis* status) was defined as positive if the animal had at least one sample culture positive confirmed by PCR and negative if hadn't reach the inclusion criteria. Due to sampling conducted at the farms, a random-effects term was included during modeling to account for extra-binominal variation attributable to lack of independence between individual animals within farms (41).

The likelihood ratio statistic was used for model development. Therefore, inclusion or exclusion of risk factors were done to test the model. Only those animals, having a complete data set were used for multivariable analysis. Rather than using a fully-saturated model containing all risk factors assessed, a starting model containing a selected subset of risk factors was utilized (41). The starting model included farm and individual-animal-level risk factors having risk ratios (RR) with a *p* ≤ 0.5 on univariable logistic-binomial regression. A forward method of variable evaluation using the likelihood ratio statistic was conducted to assess risk factor inclusion or exclusion from the final model. After a variable was added only the ones with a *p* ≤ 0.35 were kept on the model. The goodness-of-fit of the final model was evaluated by calculating the likelihood ratio statistic between the starting and final models and comparing it to the chi-square distribution. Ultimately, the most parsimonious model, was chosen to represent the data collected.

Model development (Table 2) provides summaries of herd and individual-animal-level risk factor data compiled for the 151 animals (106 cattle and 45 buffalo) involved in the study. The Generalized Linear Mixed Models (GLMM) with random effect at the individual-animal level were presented at Table 3.

**Table 3:** Generalized Linear Mixed Model with random effects of farm and individual-animal-level risk factors associated with the infection by *M. bovis* in 832 animals (106 cattle and 45 buffalo) in 41 farms in Amazonas State.

**Risk factor**	**Description**	**b**	**SE(b)**	***P*-value**	**OR**	**95% CI**
Specie	Buffalo	2.5768	1.5379	0.0968	13.15	0.623-277.28
	Cattle	0				
Herd size	Large	1.6474	1.3817	0.2358	5.19	0.336–80.399
	Medium	1.7940	1.6770	0.2872	6.01	0.216–167.20
	Small	0				
Cattle and buffalo herds	No	−1.8588	1.4870	0.2140	0.15	0.008–2.973
	Yes	0				
Random effects		4.071	2.0659			

The project obtained all necessary approvals from MSU-IRB and IACUC and from IFAM's CEPSH and CEUA.

## Results

### *M. bovis* Infection

The overall animal rate prevalence was 5.4%. At individual-animal level, a total of 151 animals (45 buffalo and 106 cattle) were considered suspect of BTB and had tissues collected for laboratory analysis, and from those a total of 45 animals (27 buffalo and 18 cattle) were confirmed by culture and PCR as positive for *M. bovis* infection. Prevalence within species was 3.0% in cattle and 11.8% in buffalo (Table 4).

**Table 4:** *M. bovis* prevalence by species in Amazonas State, Brazil.

**Species**	**N. animals inspected**	**N. of animals from which the samples were collected**	**N. of animals with Legion Suggestive of Tuberculosis (LST)**	**Positive (culture + PCR)**	**Study prevalence (%)**
Cattle	603	106	13	18	3.0
Buffalo	229	45	33	27	11.8
Total	832	151	46	45	5.40

The overall herd prevalence was 56.4%, 22 out of 39 herds had at least one animal confirmed as infected by *M. bovis*. The apparent prevalence in herds composed only by cattle, by buffalo and cattle, and only by buffalo was respectively, 45.4, 66.7, and 80% (Table 5). As reported before there were no significant differences between LST samples and no LST (42).

**Table 5:** *M. bovis* prevalence by herd in Amazonas State, Brazil.

**Herd**	Prevalence (%)^*^
Buffalo	4/5 (80%)
Cattle	10/22 (45.4%)
Buffalo and cattle	8/12 (66.7%)
TOTAL	22/39 (56.4%)

* *The herd was considered infected when it presented at least one animal confirmed positive by the PCR analysis*.

Results from the univariate logistical analysis revealed animals from dairy herds (*p* = 0.004), frequent veterinary assistance (*p* = 0.0004), and history of BTB (*p* = 0.004) were more likely to be infected with *M. bovis*. Additionally, animals that attend the floodplains (*p* = 0.001), from extensive farming systems (*p* = 0.006), and from herds with more than 100 animals (*p* = 0.05) were also more likely to be infected. Moreover, animals equal or older than 25 months were 2.7 times more likely to be infected, and buffalo and cattle living together are 2.63 times more likely to have *M. bovis* infection (Table 6).

**Table 6:** Univariate Logistic Regression of farm and individual-animal-level risk factors associated with the infection by *M. bovis* in 832 animals (106 cattle and 45 buffalo) in 39 herds in Amazonas State.

**Risk factor**	**Description**	**b**	**SE(b)**	***P*-value**	**OR**	**95% CI**
Buffalo and cattle	Yes	0.96	0.45	0.03	2.63	1.07–6.45
	No	0				
Farming	Extensive	1.01	0.37	0.006	2.76	1.33–5.71
	Semi-confined	0				
Habitat	Floodplains	1.20	0.37	0.001	3.35	1.59–7.03
	Mainland	0				
Herd age	≥25 months	0.99	0.50	0.04	2.71	1.007–7.31
	<25 months	0				
Herd health	Yes	1.98	0.56	0.0004	7.24	2.40–21.80
	No	0				
Herd size	Large	0.84	0.44	0.05	2.33	0.98–5.54
	Medium	0.44	0.43	0.96	1.55	0.65–3.68
	Small	0				
History	Yes	1.41	0.49	0.004	4.13	1.554–11.013
	No					
Purpose	Dairy	1.51	0.53	0.004	4.54	1.59–12.96
	Mix	0.66	0.45	0.14	1.93	0.79–4.68
	Beef	0				
Specie	Buffalo	1.06	0.20	0.001	8.40	3.73–18.89
	Cattle	0				

## Discussion

The observed herd prevalence 56.4% and animal rate prevalence 5.40% were the highest reported in Brazil to date (10–23). Considering only cattle, the 3.0% animal prevalence this study is the highest found in the country, where before the range was 0.04–1.3% (13, 16). It should be noted that the number of animals and herds were less than to previous studies, which may represent a limitation in this study. On the other hand, our results were based on microbiological and molecular diagnosis, while the other Brazilian studies were based only on TST screening, meaning that our results represent specificity superior to the previous studies in Brazil. In view of that if the true prevalence is the same than the observed on TST screenings, we would expect a lower prevalence than in the previous studies. Considering the sensitivity of 28.2% and specificity of 57.1% found in a controlled field study (43), the practice of TST as a screening test for BTB in Amazonas can result in a worrisome number of false-negative animals remaining in herds.

The absence of compensatory measures on the PNCEBT, is a factor to be considered as a hamper for the producers' adherence to the program, successful countries on BTB eradication adopted the screening and elimination police as well as compensatory measures to incentivize animal owners within the programs (5). Moreover, the only study found in Brazil assessing the use of TST as screening for buffaloes, found 10.81% of false positive and 33.33% of false negative on caudal fold test (CFT) and 0% of false-positive and 66.66% of false-negative on the CCT (44). These testing limitations for buffalo can represent a major challenge to elimination of BTB in Amazonas. Regardless, the observed herd and animal prevalence rates show the need for effective intervention to reduce the rates of disease in livestock populations. Additionally, it should be pointed out that there a number of slaughterhouses without inspection services in inner cities and there is a local preference for regional cheese made from raw milk. Both these practices substantially increase human exposure to *M. bovis* in Amazonas State.

This is the first BTB epidemiological study in Brazil which includes both cattle and buffalo, to our knowledge. The significantly higher prevalence in buffalo (*p* > 0.0001) agrees with previous studies (38, 39, 45). Factors that might contribute to these results can be inherent to the species, such as behavior. Buffalo are very social and commonly under pasture have a tendency to aggregation. Buffalo are also better adapted to protect themselves from the heat than cattle, in order to reduce the thermic stress, they spend lot of time wallowing in the mud, which can be a potential source of spreading *M. bovis* within the herd. This is consistent with other studies stating respiratory transmission via the inhalation of contaminated aerosols or fomites is the most efficient form of transmission, requiring few numbers of organisms as an effective dose (9, 46).

Another factor might be the differences in herd management between cattle and buffalo. Local farmers understand buffalo are more resistant to harsh environmental conditions than cattle, consequently buffalo farmers may provide less feed and routine herd health management to buffalo compared to cattle.

A third major factor to consider is genetic differences between Buffalo and cattle or related to the *M. bovis*. Are buffalo more susceptible to *M. bovis* infection than cattle? In cattle, *Bos indicus* seems to be more resistant than *Bos taurus* (11, 17, 25, 27, 47), does the same occur in buffalo? Or it may not be a host factor. Does *M. bovis* more able to infect buffalo than cattle? Studies to clarify these questions are needed. Regarding control polices, actions adequate to the reality must be in place, such as: inspection services must be more alert during inspection of buffalo carcasses in abattoir and milk in milk plants, as well as information from SIE should be used to identify infected herds.

Cattle and buffalo from large size herds were more likely to have BTB than animals from small size herds consistent with other studies conducted in Brazil (11, 12, 15, 17, 18, 22, 23) and around the world (47–53). Herd size is a major risk factor, since the number of animals in the herd increase the possibility of the transmission of the *M. bovis* increases. Moreover, in Amazonas, large herds are more commercial than small size herds, meaning that they have frequent introduction of animals from other herds and movement of animals increases BTB transmission risk within the herd. Similar results were found in the neighboring State of Rondonia (24). Modern modeling studies in England reveal that movement of infected animals was responsible for 84% of newly infected farms (31). Due to the large territory a good measure to control and eradicate the BTB should the use of the current Foot and Mouth disease zoning for implementation of a BTB zoning and implementation of control measures specifics by the zone, such as: classification of the zones according BTB prevalence, tuberculosis test requirements by zone, and movement control between the zones.

The presence of cattle and buffalo herds on the same farm increases the risk of *M. bovis* infection regardless of the specie. The presence of different livestock species increases the potential for interactions and inter-dependency among cattle and buffalo management; greater exposure leads to greater incidence. Modeling studies suggest that the environment is seriously contaminated when the practices that promote the mixing of cattle and buffalo occur, which also suggests that the cross-infection route promotes the persistence of BTB infection in cattle and buffalo populations (32). Experience in Australia showed that the complete eradication of BTB from cattle herds was possible only after the elimination of buffalo (*Bubalus bubalis*) population (5). This measure is not feasible for Brazilian circumstances, but the practices that promote the mixing of cattle and buffalo must be discouraged.

In this study, animals managed in semi-intensive and extensive systems were 52.32 and 47.68% of the sampling, respectively. Cattle and buffalo from extensive systems were 2.76 more likely to have been infected by *M. bovis* than animals raised in semi-intensive systems. This can be explained by the fact that animals in extensive systems are more likely to frequent the floodplains where multiple herds share the same pasture thereby increasing their exposure. In addition, in extensive systems, the TST and slaughter of reactors are less frequent than in semi-intensive systems. In order to determine if farming systems are influenced by other risk factors, the multivariable logistic regression demonstrated that once other factors are controlled, extensive systems are in fact protective. Although the risk factor didn't meet the eligibility criteria (*p* = 0.50) to remain in the final model, the result is coherent since semi-intensive herds are more commercial with frequent introduction of new animals from different herds and these findings agree with other studies in Brazil (12, 13, 18, 21, 23).

Based on previous studies of BTB risk factors, the purpose (milk, beef, and mix) is an important risk factor for *M. bovis* prevalence (11–13, 18, 19, 21, 23), however in this study when other risk factors are controlled the purpose of the farm wasn't significant (*p* > 0.81). The regional characterization of the farms in three categories might be an explanation for our results. The “Mix” category adopted to farms with no defined objective (milk or beef) represented almost half of the sampling and can be responsible for confounding within the model. The appropriate characterization of the farming system should be evaluated, considering other factors like breeds predominant in the herd, infrastructure, and the characteristic of neighboring herds. This may provide more accurate representation of the data for models aiming to figure better strategies to break the chain of infection of *M. bovis*.

## Conclusions

- The findings reveal an urgent need for evidence-based effective intervention aiming to reduce BTB prevalence in cattle and buffalo herds and to prevent the spread of *M. bovis* to the human population.
- Species, herd size, and production system need to be considered when developing disease surveillance and control program in Amazon.
- State zoning according the bTB prevalence and adoption of measures specific for zones is highly recommended.
- Information from Inspection Services should be used to identify infected herds.
- Practices that promote the mixing of cattle and buffalo must be discouraged.
- Studies are needed to understand why buffalo are more likely to be infected by *M. bovis* than cattle in Amazon.
- Epidemiological tools, such as modeling should be adopted for BTB control and eradication in Amazon.

This study can stimulate a discussion about the many factors potentially impacting BTB eradication schemes in Brazil and possibly stimulate new research in the areas identified.

## Data Availability Statement

The datasets generated for this study are available on request to the corresponding author.

## Ethics Statement

The animal study was reviewed and approved by MSU-IRB and IACUC and from IFAM's CEPSH and CEUA.

## Author Contributions

All authors listed have made a substantial, direct and intellectual contribution to the work, and approved it for publication.

### Conflict of Interest

The authors declare that the research was conducted in the absence of any commercial or financial relationships that could be construed as a potential conflict of interest.
